# Supplementary material for: Learning curve and surgical outcome of robotic assisted colorectal surgery with ERAS program
Source: Sci Rep. 2022 Nov 29;12:20566. doi: 10.1038/s41598-022-24665-w (PMC9709162; doi:10.1038/s41598-022-24665-w)
Supplement: Supplementary file 1 — Supplementary Information 1. [file 41598_2022_24665_MOESM1_ESM.zip › LSC Raw data-Ñ[▒K/134/O_ñw╝╨░O▒Kñσ.pdf]

正式摘要(電子簽章：0000002236133881)

W83 / W83

臺中榮民總醫院

Operation Note

[redacted]

- 手術日期：2021/08/26
- 手術醫師：蔣鋒帆，Fellow 楊曙榮，R 洪浚嚴，R 蘇晏申
- Anesthesia：GE
- Position：Supine
- Aseptic agents：Soap，Betadine Alcohol，Betadine Aqua

Diagnosis

- Pre-operative diagnosis : HEPATCI FLEXURE CA PROVED
- Post-operative diagnosis : Ditto. s/p right hemicolectomy with side to side ileocolonal anastomosis

Surgical Indication

- for radical resection and pathologic staging

Operation

- 1. Laparoscopy convert to open due to advanced tumor, Right hemicolectomy with side-to-side anastomosis, Barcelona method with GIA 75mm*2, re-inforcement with silk (+).
  2. Vessel ligation : ileo-colic, right colic, and right branch of middle colic vessels
  3. Repair of mesentery defect: nil.
  4. cholecystectomy was done due to tumor severe adhision to Gall bladder
  5. Surgiwrap +

Finding

- 1.One hudge tumor, measured about 8 x 10 cm in size, located at hepatic flexture colon, which invaded to Gall bladder
  2.No other gross tumor or polyp was found.
  3.There was no visible liver metastasis or visible peritoneal seeding.
  4.The duodenum was intact.
  5.Some cloudy whitish ascited noted

Procedure

- General endotracheal anesthesia was induced and then the patient was placed in the supine position. Preoperative antibiotics were given. A Foley catheter and nasogastric tube were placed. The abdomen was prepped and draped in standard sterile fashion. Laparoscopy convert to open due to advanced tumor. A vertical midline incision was made and deepened through the subcutaneous tissues and hemostasis was achieved with electrocautery. The linea alba was identified and incised and the peritoneal cavity entered. The operative findings were as aforementioned. The small bowel was inspected and retracted to the left using a moist pad and self-retaining Balfour retractor. Using electrocautery, the colon was freed from its peritoneal attachments along the line of Toldt from the cecum to the hepatic flexure. The duodenum was identified and protected, as were the pancreatic head. The peritoneum overlying the mesentery was then scored with electrocautery and the ileocolic artery was identified, ligated with double 2-0 silks, and transected. The right colic vessels and right branch of middle colic vessels were also divided and ligated near the root. Hemostais was checked in the operative field. Cecum, ascending colon and hepatic flexure were mobilized and points of transection were selected proximally and distally. Side to side anastomosis was done Barcelona method, GIA 75-3.5mm*2. Lembert's sutures were done. The anastomosis was checked and found to be intact and widely patent. The abdomen cavity was then copiously irrigated and hemostasis was checked. The fascia was closed with No.1 Vicryl. The

- skin was closed wtih 3-O Nylon. The patient tolerated the procedure well and was brought to the POR under stable condition.
- Estimated blood loss: minimum
- 器械數量: 正確
- 紗布數量: 正確

Image

- PAINT
    - abdomen

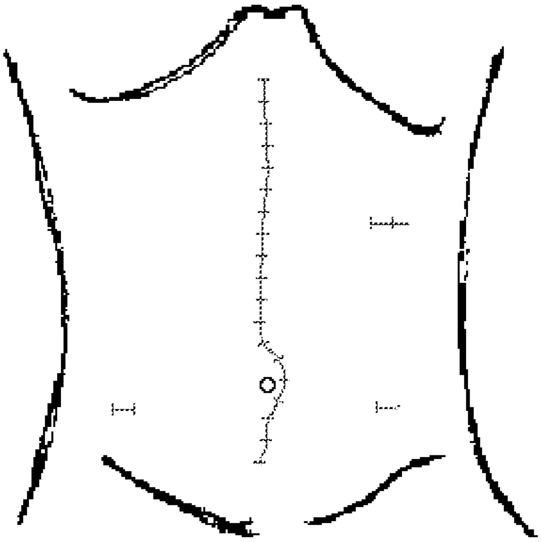

    - colon & rectum

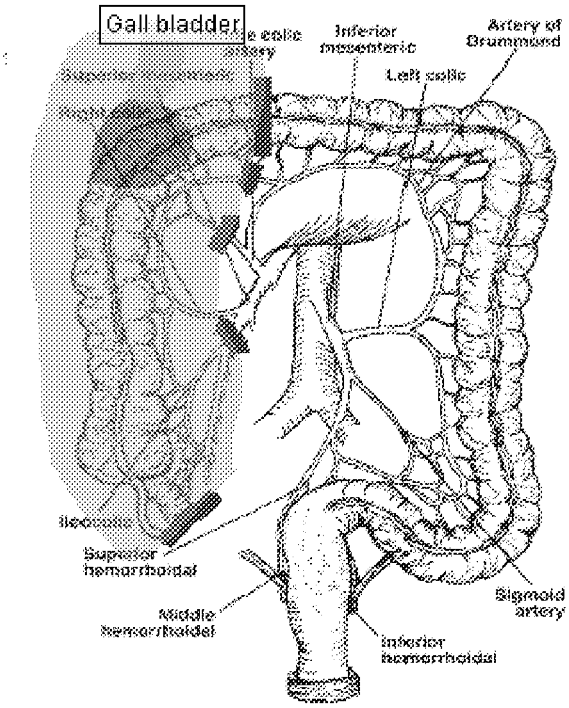

主任 2062F蔣鋒帆 / 醫師 2292G楊曙榮

正式摘要(電子簽章：**000000223613881)**

W83 / W83

doctorRank:B
